# Acoustic Divergence with Gene Flow in a Lekking Hummingbird with Complex Songs

**DOI:** 10.1371/journal.pone.0109241

**Published:** 2014-10-01

**Authors:** Clementina González, Juan Francisco Ornelas

**Affiliations:** Departamento de Biología Evolutiva, Instituto de Ecología AC, Xalapa, Veracruz, México; Utrecht University, Netherlands

## Abstract

Hummingbirds have developed a remarkable diversity of learned vocalizations, from single-note songs to phonologically and syntactically complex songs. In this study we evaluated if geographic song variation of wedge-tailed sabrewings (*Campylopterus curvipennis*) is correlated with genetic divergence, and examined processes that explain best the origin of intraspecific song variation. We contrasted estimates of genetic differentiation, genetic structure, and gene flow across leks from microsatellite loci of wedge-tailed sabrewings with measures for acoustic signals involved in mating derived from recordings of males singing at leks throughout eastern Mexico. We found a strong acoustic structure across leks and geography, where lek members had an exclusive assemblage of syllable types, differed in spectral and temporal measurements of song, and song sharing decreased with geographic distance. However, neutral genetic and song divergence were not correlated, and measures of genetic differentiation and migration estimates indicated gene flow across leks. The persistence of acoustic structuring in wedge-tailed sabrewings may thus best be explained by stochastic processes across leks, in which intraspecific vocal variation is maintained in the absence of genetic differentiation by postdispersal learning and social conditions, and by geographical isolation due to the accumulation of small differences, producing most dramatic changes between populations further apart.

## Introduction

Understanding the processes and mechanisms by which phenotypic diversity arises is fundamental in evolutionary biology. Although most of research has focused in the role that natural selection can play in the processes of population divergence, an interest to study the role of stochastic processes involved in phenotypic and population divergence has raised lately [1]–[5]. Divergence in acoustic signals is particularly interesting because population differences can accrue over short evolutionary timescales through learning and cultural evolution [3], and because the rapid change of a signal-receiver communication system could reduce gene flow between populations, promoting neutral genetic differentiation [6]–[10]. Intraspecific geographic variation in acoustic mating signals or dialects can potentially facilitate reproductive isolation through assortative mating (females that prefer local male signals to foreign ones; [11]) and, if so, a reduction in dispersal among dialects can be observed, affecting success in finding mates and/or access to social groups [12].

Song divergence among populations can be driven by three non-mutually exclusive processes: (1) selection of particular acoustic traits due to environmental or habitat characteristics, where different frequencies of sound travel best in different environments or because ambient noise differences between environments [9], [13]–[19]; (2) sexual, social and cultural selection, where this kind of selection might cause rapid evolution of acoustic signals involved in mating because the attractiveness of novelty, the potential for runaway change and the absence of well-defined optima [20]–[21]; and (3) genetic and cultural drift, where song divergence occurs in the absence of any kind of selection [22]–[24]. Studies relating intraspecific genetic divergence and acoustic mating signals have featured mainly oscine passerines (*e.g.*, [18], [21],[25]), a suboscine passerine [26], and parrots (*e.g.*, [12]), but hummingbirds in which vocal learning has evolved independently [27]–[28] have been rarely studied [14]. Like parrots and songbirds, hummingbirds have also developed the trait of vocal learning [27], and although they sing apparently single-note songs [29]–[31], there are species intermediate in vocal complexity [30], [32]–[33], and species rivaling passerines with intricate, phonologically and syntactically complex songs [14], [27], [30], [34]–[38].

The wedge-tailed sabrewing, *Campylopterus curvipennis* (Deppe, 1830), is a sexually monochromatic, size dimorphic hummingbird with a wide distribution through the cloud forest and humid tropical forest of eastern Mexico [14], [39]. Males are polygynous and during the breeding season they congregate in leks, broadcasting elaborate vocalizations into the environment [36]. Males attending leks are highly phylopatric (C. González and J.F. Ornelas, unpublished data), and their songs are loud, high-pitched, and are composed of several discrete units (syllables), with a highly variable and complex acoustic structure [36]. The wedge-tailed sabrewings display songs with many levels of geographic variation, most notably the introductory syllable and the syllable repertoire, from differences between neighboring males within a lek (song neighborhoods; [37]), differences between lek members separated by few kilometers [36] to differences between geographically isolated subspecies [14]. These characteristics offer an interesting model for the study of signal evolution and song elaboration among lek breeders and for the understanding of the origin and maintenance of song geographic variation.

A recent broad-scale phylogeographic study [14] revealed three main results. First, *Campylopterus curvipennis* is a species complex composed of three allopatrically distributed lineages corresponding to subspecies *C. c. curvipennis* along the Sierra Madre Oriental, *C. c. excellens* in the Sierra de los Tuxtlas, and *C. c. pampa* in the Yucatan Peninsula. Second, morphological and acoustic data along with information about the habitat (climate and topography) indicated that these subspecies are morphologically and acoustically divergent, independently of habitat-related variation, and that the genetic divergence among subspecies was coupled with song divergence. Third, a coalescent analysis to evaluate whether song divergence could be attributed to drift or selection, suggested that the fixation of song types, assumed to be encoded by nuclear genes, has occurred faster than expected by genetic drift, suggesting that selection may have played a role in driving song evolution in the past. However, the assumption that fixation of song types is encoded by nuclear genes needs to be taken with caution in such a dynamic cultural system. Furthermore, the observed positive relationship between genetic and acoustic distances in that study, independent of geographic distances, is expected under a drift model of song evolution, where song divergence is higher between allopatric populations that have been isolated for the longest time (i.e. subspecies) with low potential for hybridization ([14] see also [18], [40]). Lastly, selection-driven genetic evolution can be confounded when cultural drift occurs faster than genetic drift, or the association between genetic and acoustic divergence generated by founder effects and/or allopatric genetic and cultural evolution [8], [41].

Here we examine geographic variation in song features of males across leks distributed at a smaller geographical scale without apparent isolating barriers to assess the role of non-geographic stochastic processes in shaping lek differences in song. Specifically, we quantify male song variation in the subspecies *curvipennis* across leks located along the Sierra Madre Oriental, and ask what processes best explain the observed patterns. We then derived estimates of lek genetic structuring from microsatellite loci to be contrasted with measures of song divergence. Our study examines three main hypotheses proposed to explain the evolutionary significance, if any, of geographic song variation (reviewed in [41]). Briefly, the genetic adaptation hypothesis [42]–[44] posits that gene flow among conspecific populations can be reduced to some degree in the presence of song dialects if young birds learn to produce or recognize song while still in their natal region, and that adults use song as a cue for assortative mating. Given these conditions, the spatial distribution of song dialects should mirror the genetic structure of populations. Similarly, the social adaptation hypothesis [45] proposes that song dialect influences social and sexual interactions, but modification of male songs in adulthood to match those of their neighbors does not produce genetic isolation of dialect groups. In contrast, the epiphenomenon hypothesis [46] suggests that dialect formation is mere by-product of vocal learning. This hypothesis predicts genetic isolation of dialect regions, but such isolation is due to neutral processes such as founder effects or geographic barriers, rather than to assortative mating based on song [41]. One prediction of all three hypotheses relates to the role of dialects in the genetic structuring of populations. The social adaptation hypothesis predicts no relationship between song dialects and the genetic structure of populations, whereas the genetic adaptation hypothesis predicts that dialects affect the genetic structure of populations, and the epiphenomenon hypothesis predicts that dialects may be associated with genetic structure of populations (although not causally; reviewed in [41]). Given that the association between genetic and acoustic divergence at the subspecific level [14] is confounded by the geographic isolation between subspecies, a strong coupling between genetic and acoustic divergence is expected at a lower distributional scale (within subspecies), as predicted by the adaptation and epiphenomenon hypotheses, in which no apparent geographic or habitat-related barriers exist.

## Materials and Methods

### Ethics Statement

We obtained the collecting permit to conduct this work from the Mexico’s Secretaría de Medio Ambiente y Recursos Naturales, Instituto Nacional de Ecología, Dirección General de Vida Silvestre (permit number: INE, SEMARNAT, SGPA/DGVS/02038/07) for the field study described. This collecting permit specifically allowed for the collection of tail feathers from the birds. Manipulation of birds in the field was minimal. Birds were captured at leks with mist nets and their two outermost tail feathers were removed for genetic analyses before they were released. All procedures with birds were carried out in accordance with the Guidelines for the Use of Wild Birds in Research proposed by the North American Ornithological Council and the ethics of experimental procedures were revised and authorized by the Animal Care and Use Committee under the Graduate Studies Committee (Doctorado en Ciencias Biomédicas) of the Universidad Nacional Autónoma de México (UNAM), the Instituto de Ecología, A.C. (INECOL), and CONACyT (61710). While the field studies involve a non-threatened or protected species, no specific permits are required for song recording or field studies as the one described here.

### Sample Collection and DNA Sequencing

Feather samples were collected from a total of 105 wedge-tailed sabrewings captured during the 2006–2008 breeding seasons (**Table S1**), with 3–27 individuals sampled per lek. Sampled leks along the Sierra Madre Oriental (SMO) were categorized in three geographic areas: (i) northern limit of their distribution, from southern Tamaulipas and north of San Luis Potosí (nSMO herein, n = 3 leks); (ii) central, from south of San Luis Potosí (cSMO herein, n = 2 leks); and (iii) southern limit of the distribution, from Puebla and Veracruz (sSMO herein, n = 4 leks) (**Table S1**).

### Microsatellites Genotyping

DNA extraction was made from the calamus of one of the two removed feathers with chelex (5%) according to Morin et al. [47], and samples were genotyped at 10 polymorphic microsatellite loci designed specifically for *Campylopterus curvipennis* ([48], GenBank accession nos. GQ294539–GQ294550). A full description of the development protocol for the loci, PCR conditions, and fragment sizing of microsatellites can be found at the Molecular Ecology Resources Database (http://tomato.biol.trinity.edu/; [48]). Observed and expected heterozygosity and mean number of alleles per locus were calculated in GENEPOP v3.4 [49]. Microsatellite genotypes were tested for departures from Hardy-Weinberg equilibrium and for linkage disequilibrium between pairs of loci within leks in GENEPOP. The presence of null alleles was tested using MICROCHECKER v2.2.3 [50]. MICROCHECKER infers the presence of a null allele when significant excess homozygosity is distributed evenly across all of the alleles at a locus.

### Genetic Analysis

To investigate lek genetic structure we calculated global and pairwise R_ST_ [51] and F_ST_ parameters of genetic differentiation due to genetic structure in RSTCALC v2.2 [52] and FSTAT v2.9.3 [53] respectively. To examine geographic patterns of population genetic structure, we performed Bayesian genetic clustering using STRUCTURE v2.2.3 [54] to infer the most likely number of genetic clusters (*K*) based on microsatellite data. We ran two independent STRUCTURE analyses, with the LOCPRIOR or the default mode, both under the admixture model with correlated allele frequencies [54]. The default mode for STRUCTURE uses only genetic information to assign individuals into clusters, whereas the LOCPRIOR mode uses sampling locations as prior information to assist the clustering-for use with data sets where the signal of structure is relatively weak [54]. Ten independent chains were run for each *K*, from *K* = 1 to *K* = 12. The length of the burn-in was 500,000 and the number of Markov chain Monte Carlo (MCMC) replications after the burn-in was 1,000,000. To determine an accurate number of clusters we calculated the statistic Δ*K* based on the rate of change in the log probability of data between successive *K* values following Evanno et al. [55].

Gene flow (4*N_e_m*) between leks and between groups of leks (nSMO, cSMO, sSMO) was estimated with a maximum likelihood coalescent approach using genotypic data and MIGRATE v3.0 [56]. The first genealogy was started with a random tree, and initial theta and migration rate (xNm) parameters were obtained from F_ST_ calculations. We ran five short and three long chains (200,000 genealogies sampled) or 20 short and six long chains (500,000 genealogies sampled) for estimation between leks and between groups of leks, respectively, after discarding the first 5,000 genealogies as a burn-in.

### Acoustic Analysis

Songs were recorded from territory-holding males at the same leks in which feather samples were taken. Between 3 and 13 birds were recorded at each lek, with 5 to 19 song recordings obtained from each of 56 males (500 recordings, *c.* 9 recordings per bird). Some of these recordings were used in previous studies [14], [37]. Song recordings were made with a Marantz PMD660 portable solid-state recorder and a Sennheiser MKH-70 directional microphone. Recordings were digitized at a sampling rate of 44100 Hz and stored as 16-bit samples. Spectrograms of recordings from all individuals were generated with a 349.7 Hz filter bandwidth and a frame length of 512 points ( = 11.6 ms) using RAVEN v1.4 (www.birds.cornell.edu/raven).

**Figure 1:**
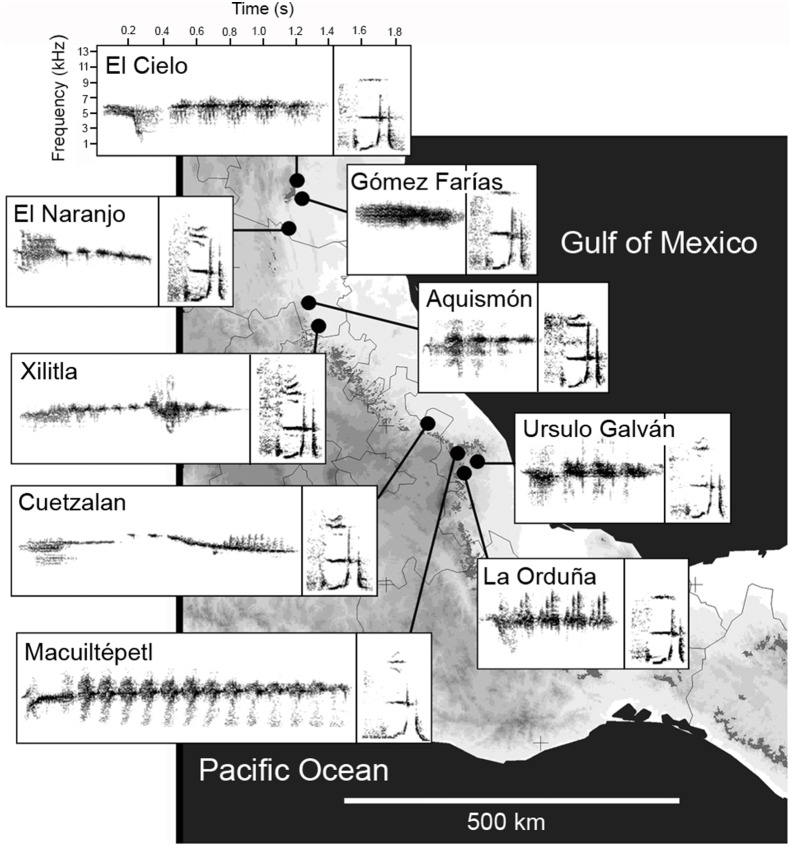
Spectrograms of introductory (left) and shared syllable (right) placed on to a map of the geographic distribution of leks. Both syllables are typical to each lek, most notably the introductory syllable, and the 70 and 82% of the individuals were correctly classified to their lek of origin according to measurements from the introductory and shared syllable, respectively. See Fig. S1 for further details on measurements taken from the introductory and shared syllable.

Based on the digitized songs we constructed three different data sets for acoustic analyses: (1) presence/absence of syllable types, (2) relative frequency of each syllable per song, and (3) acoustic traits of two comparable syllables. For the first two data sets, we used syllabic units throughout the wedge-tailed sabrewing repertoire. Syllables were visually classified by CG based on the structure observed on the printed spectrograms, and assigned a letter or combination of letters for further identification. A syllable was defined as an element or several elements always grouped together in a fixed composition. The visual classification of syllable types was not difficult, as syllables are discrete units composed of one or more elements that in general differ consistently in their acoustic structure in obvious ways (for details see [37]). As the spectrograms were generated, each syllable was distinguished from each other by visually inspecting the acoustic characteristics of syllables (shape and duration, frequency of inflection points, presence and number of harmonics) and by listening the syllables at low speed. The visual inspection of spectrograms and visual classification of syllable types were done without knowledge of the outcome of the analyses. On average, we detected 34 syllable types emitted per individual across leks. Our sampling based on the number of syllable types was reasonably complete as the repertoire of a male reached an asymptote of *c.* 30 syllable types with *c.* 10 songs recorded [36]. We screened the 500 recordings and built a presence/absence matrix of syllable types for each of the 56 individuals, where each entry consists of 0 or 1, and a matrix with the relative frequency of each syllable per song (number of times that a syllable is sung), averaged by bird. For the third data set, we took spectral and temporal measurements of two syllables to allow comparisons: the introductory syllable emitted by all individuals at the beginning of the song bout, and a syllable in the repertoire shared by all recorded individuals across leks emitted at least once in the songs (**Figure 1**, **Figure S1**). For the introductory syllable we quantified the total number of elements, and the number of different elements. For the introductory and the shared syllable, we measured the duration (s), minimum frequency (kHz), bandwidth (the difference between maximum and minimum frequency, kHz), and peak frequency (frequency at which maximum power occurs, kHz; **Figure S1**). Because the shared syllable has several elements (notes), we took the same measures on two of their elements (numbers 1 and 2, **Figure S1**) and the duration of another element (number 3, **Figure S1**). In total six measures were taken on the introductory and 13 on the shared syllable.

To evaluate song divergence among leks, we performed a hierarchical cluster analysis using the unweight pair-group method with arithmetic mean (UPGMA) and the Euclidean distances to construct a dendrogram in SPSS (SPSS Inc.) based on the binary presence/absence matrix of syllable types. We additionally ran STRUCTURE v2.2.3 [54] to graphically represent geographic patterns of acoustic structure and compare these results with those obtained for genetic structure. The Bayesian clustering technique has been usually applied to most commonly used genetic markers, however, STRUCTURE is also applicable to analyses based on phenotypic data, such as analysis of human language [57]. We used the presence/absence matrix of the syllable types (where each syllable type is treated as a different locus, and the values 0 or 1 is the equivalent of the genetic alleles) to determine the most likely number of acoustic clusters (*K*). The ploidy option was set at 1, and ten independent chains were run for each *K*, from *K* = 1 to *K* = 12. Length of the burn-in was 250,000 and the number of Markov chain Monte Carlo (MCMC) replications after the burn-in was 500,000. To determine the accurate number of clusters, Δ*K* was also calculated.

We used a principal components analysis (PCA) with the measurements of the introductory and the shared syllable to reduce the number and intercorrelation of the variables. The resulting PC scores were tested for significant differences among leks and between groups of leks (see Results) performing multivariate analyses of variance (MANOVA) followed by one-way ANOVAs. Mensural data and counts were log- and square root transformed, respectively, to produce normality in the data. Finally, we used the relative frequency of syllables, and the measurements of the introductory and shared syllable as predictors in a discriminant function analysis (DFA) for each of the three data sets to examine whether individuals could be classified according to their lek of origin.

### Comparison between Acoustic, Genetic and Geographic Distances

Acoustic distance matrices regarding song sharing were built by estimating the pairwise Jaccard similarity coefficient between individuals among leks. This coefficient was calculated in ESTIMATES v8.0.0 [58] and values were subtracted from 1 to make a dissimilarity matrix. Distance matrices for the introductory syllable and the shared syllable measurements were built as the Euclidean distance between the group centroids of first discriminant functions using SPSS, in which variables were scaled to values between 0 and 1. We followed Ruegg et al. [18] to examine relationships between acoustic, genetic and geographic distances. Namely, we performed a series of simple and partial Mantel tests using IBD v3.23 [59], assessing significance levels of association between matrices with 1000 randomizations. To test the prediction that song divergence is positively correlated with genetic divergence, we compared distances of the song sharing and the introductory and shared syllable with pairwise R_ST_ and F_ST_ distances. These relationships were controlled for potential effects of geographic distance through partial Mantel tests. To test for the effects of isolation by distance we looked at the relationships between acoustic and geographic distance, controlling for genetic distance. Finally, we tested the relationships between genetic and geographic distances.

## Results

### Genetic Structure among Leks

The number of alleles per locus varied from 3 to 15, and the observed heterozygosity values indicate no consistent deviations from H-W equilibrium. After Bonferroni corrections, only four leks deviated from H-W equilibrium in locus CACU13-2, two in locus CACU5-7, and one lek in locus CACU13-7 (**Table 1**). MICROCHECKER identified that these loci were possibly affected by the presence of null alleles. No significant linkage disequilibrium was detected in any of the population-loci comparisons after Bonferroni corrections.

**Table 1:** Population genetic variability in wedge-tailed sabrewing leks based on ten microsatellite loci.

Lek	*n*	Mean alleles/locus	*H*_O_	*H*_E_
El Cielo	18	5.6	0.47*	0.59
Gomez Farías	4	3.6	0.65	0.61
El Naranjo	6	3.6	0.55	0.57
Aquismón	4	3.5	0.55	0.63
Xilitla	8	5	0.49*	0.64
Cuetzalan	27	6.2	0.57	0.62
Macuiltépetl	3	2.66	0.55	0.57
La Orduña	22	6.2	0.49*	0.63
Ursulo Galván	13	5.3	0.48* †ζ	0.60

*n* = sample size, *H*_O_ = observed, and *H*_E_ = expected heterozygosity;

*indicates significant departure (*p*<0.05, after sequential Bonferroni correction) from Hardy-Weinberg equilibrium for locus CACU13-2,

† for locus CACU13-7, and ζ for locus CACU5-7.

**Figure 2:**
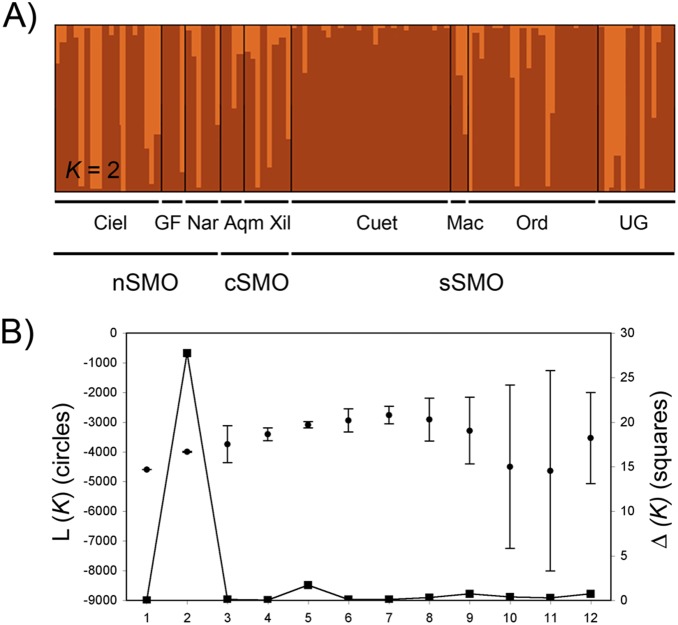
Geographic patterns of genetic structure based on Bayesian assignment analysis in STRUCTURE. (A) Posterior assignment probabilities of 105 individuals of *C. curvipennis* show a weak genetic structure, with an optimal number of *K* = 2. Each individual is represented by a vertical line that is partitioned into *K* colored sections, with the length of each section proportional to the estimated membership coefficient. (B) Mean log probability of the data (L(*K*) ± SD) over 10 independent runs from *K* = 1 to *K* = 12 (left Y axis) and values of Δ*K* calculated according to Evanno et al. [55] (right Y axis).

We did not detect significant genetic subdivision among leks (global R_ST_ estimate ± SE, –0.032±0.0017, *p* = 0.455; global F_ST_ estimate ± SE, 0.021±0.002, *p* = 0.972). Pairwise R_ST_ among leks ranged from –0.053 to 0.082, and pairwise F_ST_ ranged from –0.0138 to 0.249. Results of STRUCTURE analysis showed no genetic clustering when population information was not provided, whereas a weak genetic structure among leks was revealed when using the LOCPRIOR function. When Δ*K* was calculated the break in slope of the distribution of L(*K*) was at *K* = 2 (**Figure 2**). At this optimum we did not observe a clear pattern of genetic clustering with a lek or a geographic correspondence because they have an admixed ancestry.

Estimates of gene flow among leks along the Sierra Madre Oriental were heterogeneous and highly asymmetric (**Table 2**) without a clear geographic pattern of gene flow. For example, gene flow values from all leks to the Ciel lek, which is in the northern limit of the distribution, were much lower than one. The Cuet lek seems to generate more emigrants per generation but receive few immigrants. Finally, Aqm and Mac leks had the highest of all estimates of gene flow, however, these estimates of gene flow can be overestimated given the lower sample size of these leks. Interestingly, estimates of gene flow between groups of leks were increasingly greater from north to south (nSMO → cSMO = 0.008, nSMO → sSMO = 1. 788, cSMO → sSMO = 3.387), but vanishingly small to zero in the opposite direction (sSMO → cSMO = 0.029, sSMO → nSMO = 0, cSMO → nSMO = 0.0004).

**Table 2:** Estimates of the gene flow parameter 4 Nm generated in Migrate analysis among leks from 10 microsatellite genotypes.

	Ciel	GF	Nar	Aqm	Xil	Cuet	Mac	Ord	UG
Ciel	––	3.58	0.58	17.62	6.23	0.25	69.2	0.32	1.4
		(2.24–5.32)	(0.28–1)	(12.96–23.24)	(4.76–7.96)	(0.14–0.4)	(46.2–98.7)	(0.18–0.52)	(0.78–1.6)
GF	0.05	––	0.16	4.95	0.15	0.05	10.93	0.24	0.22
	(0.004–0.17)		(0.04–0.44)	(2.71–8.2)	(0.02–0.55)	(0.01–0.13)	(3.52–24.8)	(0.01–0.17)	(0.08–0.44)
Nar	0.09	1.19	––	1.65	0.3	0.1	10.93	0.19	0.27
	(0.02–0.25)	(0.48–2.28)		(0.53–3.76)	(0.07–0.8)	(0.04–0.21)	(3.52–24.8)	(0.09–0.35)	(0.12–0.52)
Aqm	0.14	0.24	0.25	––	0.46	0.05	3.64	0	0.11
	(0.04–0.32)	(0.24–0.88)	(0.08–0.56)		(0.14–1.04)	(0.01–0.13)	(0.38–13.28)	(0–0.51)	(0.03–0.29)
Xil	0.37	1.91	0.66	2.75	––	0.07	14.57	0.06	0
	(0.2–0.64)	(1–3.24)	(0.34–1.12)	(1.19–5.28)		(0.02–0.17)	(5.6–30.04)	(0.01–0.17)	(0–0.07)
Cuet	1.31	4.53	1.81	11.01	4.26	––	58.28	1	0.71
	(0.92–1.76)	(3.04–6.48)	(1.24–2.52)	(7.44–15.56)	(3.06–5.72)		(37.44–85.6)	(0.73–1.32)	(0.43–1.08)
Mac	0.14	0.48	0	0.55	0.46	0.02	––	0.06	0
	(0.04–0.32)	(0.11–1.28)	(0–0.11)	(0.06–2.0)	(0.14–1.04)	(0.00–0.09)		(0.01–0.17)	(0–0.07)
Ord	0.61	2.15	1.11	5.5	0.61	0.68	61.92	––	0.43
	(0.36–0.92)	(1.16–3.56)	(0.64–1.64)	(3.12–8.88)	(0.23–1.25)	(0.49–0.92)	(40.36–90)		(0.23–0.74)
UG	0.19	1.67	0.91	2.75	1.82	0.33	21.49	0	––
	(0.07–0.36)	(0.84–2.92)	(0.52–1.64)	(1.19–5.28)	(1.08–2.83)	(0.2–0.92)	(12.72–44.8)	(0–0.04)	

Donor populations are on horizontal, recipient populations are on the vertical. Estimates are given followed by 95% confidence intervals in parentheses.

### Acoustic Variation and Song Divergence

**Figure 3:**
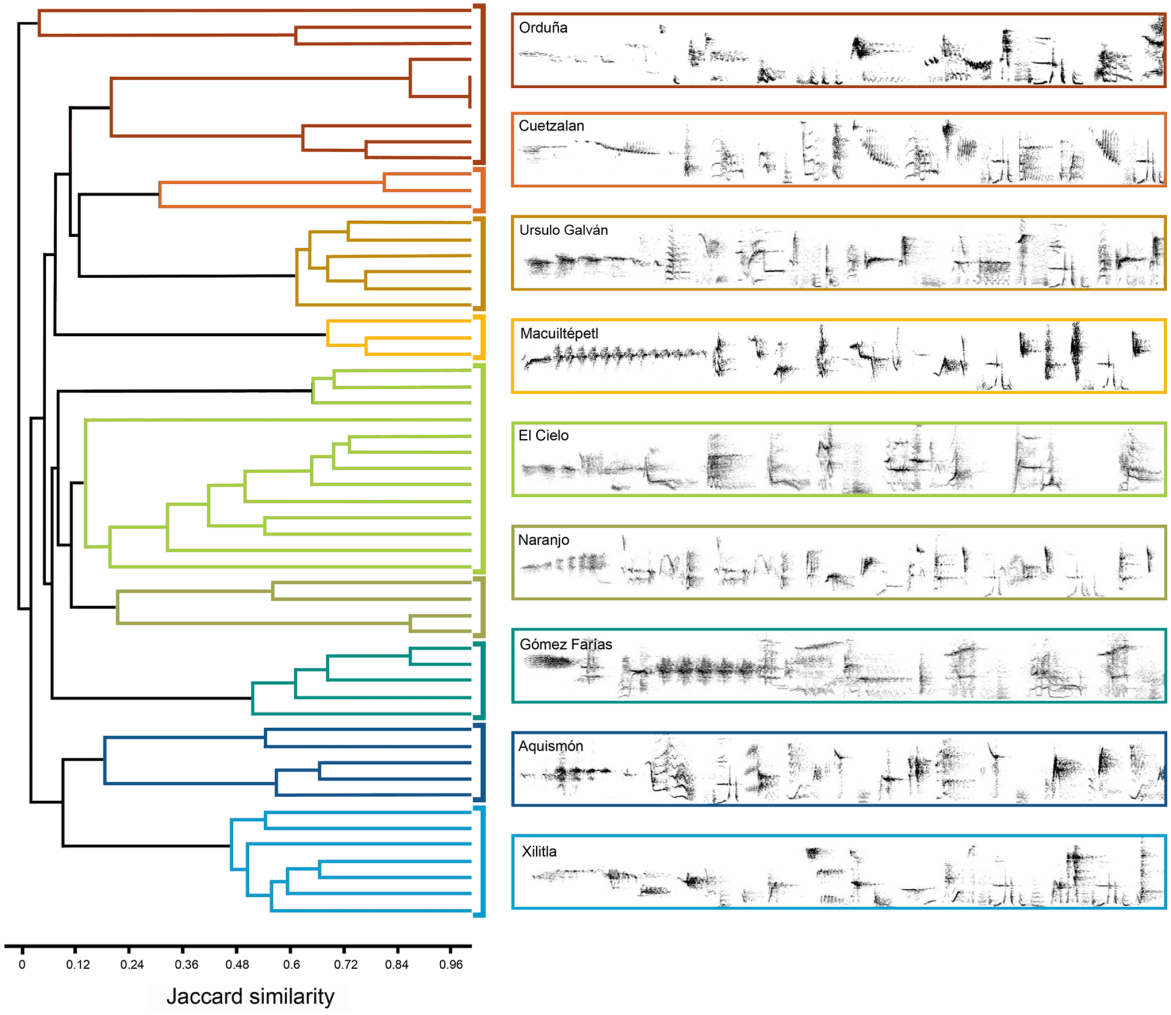
Dendrogram generated by cluster analysis of a presence/absence matrix of syllable types from individuals recorded. Each of nine leks is located in a different cluster and represented by a different colored line. Green, blue and red-orange lines correspond to leks from the north, central and south part of the Sierra Madre Oriental respectively (nSMO, cSMO, and sSMO). Attached to the dendrogram, 4 sec fragments of vocalizations representing syllable variation of each lek are shown. Audio files of one song of each lek are provided as (**Audio S1–S9**).

In total we detected 294 syllable types across males recorded at leks. The dendrogram derived from the cluster analysis based on the presence/absence of syllable types showed that individuals within the same lek are acoustically more similar than those between leks, except those from the Ciel and Ord leks (**Figure 3** and **Audio Files S1–S9**). In addition, we observed a geographic pattern in song sharing: leks from the northern (GF, Ciel, Nar; nSMO), central (Aqm and Xil; cSMO) and southern limit of the distribution (Cuet, Ord, UG and Mac; sSMO) are clustered together in different groups, except three individuals from the Orduña lek, which are basal in the dendrogram. On average, the proportion of syllables shared between individuals within leks was 0.60±0.18 (mean±SD), whereas the proportion of syllables shared among leks was 0.15±0.04, these correspond to 20.4 and 5.1 syllables respectively (**Table 3**). Despite the great syllable diversity observed across leks, there were some syllables extensively shared, whereas only members from closely distributed leks shared others.

**Table 3:** Similarity matrix based on the presence/absence of syllable types among leks.

Lek	Ciel	GF	Nar	Aqm	Xil	Cuet	Mac	Ord	UG
Ciel	**0.437**								
GF	0.191	**0.771**							
Nar	0.218	0.189	**0.481**						
Aqm	0.144	0.144	0.163	**0.471**					
Xil	0.112	0.111	0.178	0.199	**0.673**				
Cuet	0.102	0.126	0.129	0.119	0.109	**0.459**			
Mac	0.099	0.125	0.111	0.091	0.099	0.090	**0.840**		
Ord	0.174	0.190	0.203	0.135	0.120	0.146	0.162	**0.424**	
UG	0.175	0.160	0.214	0.139	0.176	0.176	0.137	0.235	**0.823**

Values in bold indicate comparisons between individuals within the same lek.

**Figure 4:**
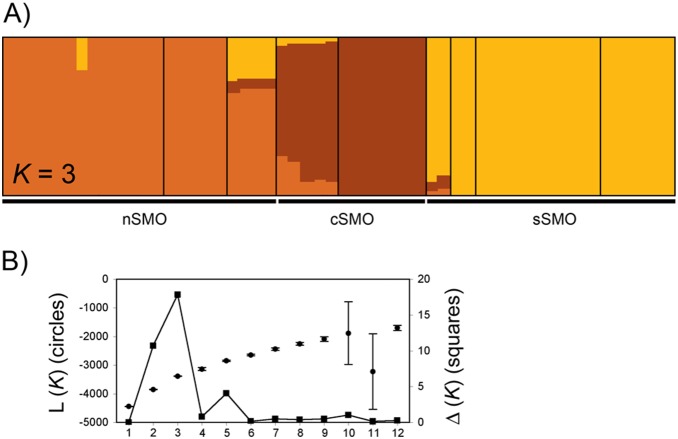
Geographic patterns of acoustic structure based on Bayesian assignment analysis in STRUCTURE using the presence/absence matrix of the syllable types. (A) Posterior assignment probabilities of 56 individuals of *C. curvipennis* show the uppermost level of structure with the maximum probability at *K* = 3 corresponding to the northern, central and southern areas of distribution. Each individual is represented by a vertical line that is partitioned into *K* colored sections, with the length of each section proportional to the estimated membership coefficient. (B) Mean log probability of the data (L(*K*) ± SD) over 10 independent runs from *K* = 1 to *K* = 12 (left Y axis) and values of Δ*K* calculated according to Evanno et al. [55] (right Y axis).

**Figure 5:**
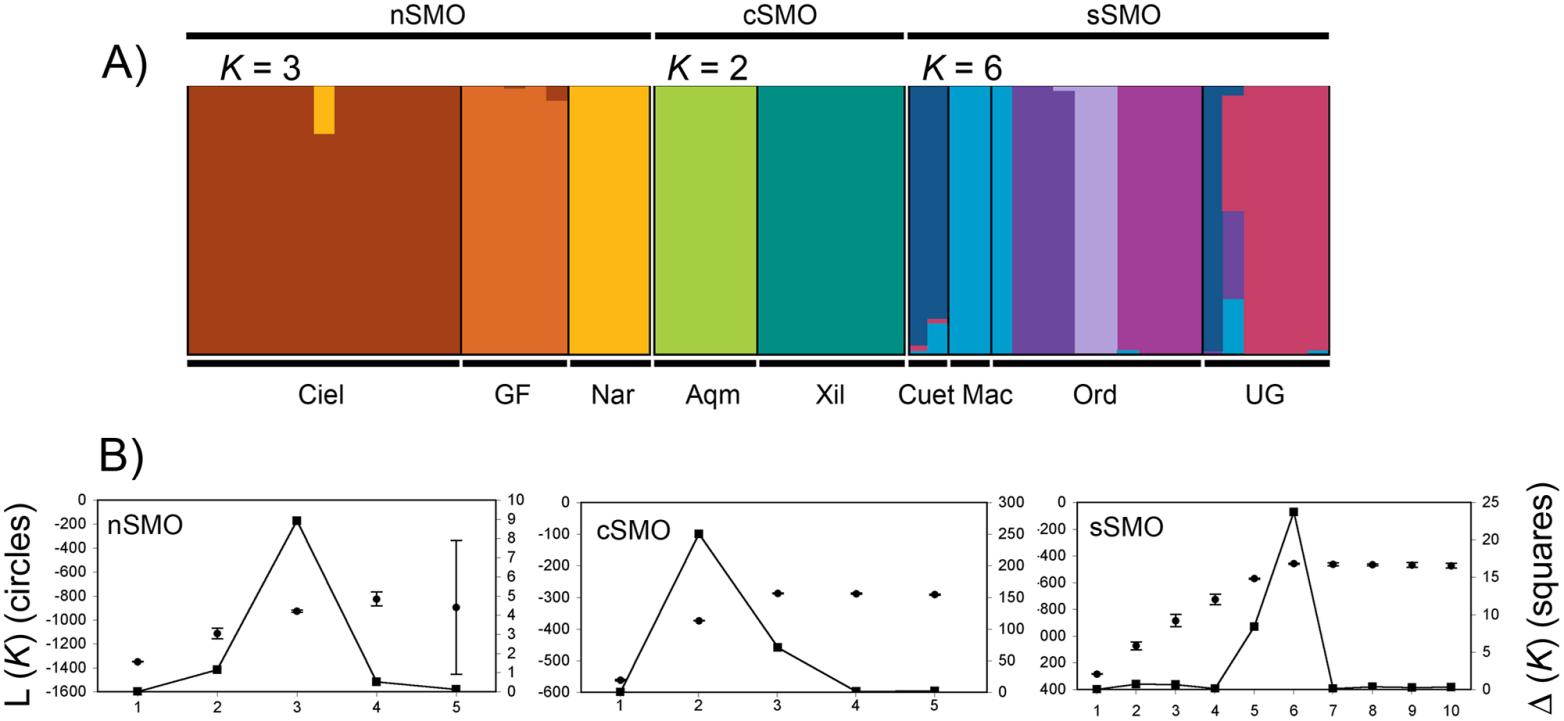
Additional independent STRUCTURE analyses with each subset of data of the three clusters detected, showing a strong acoustic structure. (A) Posterior assignment probabilities showed where optimal number of cluster were *K* = 3 for nSMO, *K* = 2 for cSMO and *K* = 6 for sSMO. B) Mean log probability of each data set (L(*K*) ± SD) according to Evanno et al. [55].

The results of STRUCTURE showed a strong pattern of syllabic structure. The break in the slope of the distribution of L(*K*) was at *K* = 3 when Δ*K* is calculated (**Figure 4**). Again, the three clusters correspond to leks distributed in the northern, central and southern part of the geographic distribution. As the Δ*K* method finds the uppermost level of structure [55], we conducted subsequent analyses on subsets of the data using the three clusters identified by STRUCTURE to determine whether any additional substructure was detected. For these analyses STRUCTURE was run for each subset with ten independent chains for each *K*, from *K* = 1 to *K* = 5 for nSMO and cSMO and from *K* = 1 to *K* = 10 for sSMO. For the nSMO cluster Δ*K* was the highest at *K* = 3, and for the cSMO cluster Δ*K* was the highest at *K* = 2 (**Figure 5**). In both analyses the number of *K* clusters corresponds to the number of leks in each subset. In the case of sSMO Δ*K* was the highest at *K* = 6 (**Figure 5**). Here, all leks are assigned probabilistically to different clusters, except some Cuet individuals assigned to the UG lek and some Mac to the Ord lek. Overall these results indicated a hierarchical structure in the acoustic data sets, where three principal clusters are detected in the first hierarchical level of the analysis, but when analyzed each subset of data, individuals are probabilistically assigned to the lek they belong.

The six measurements of the introductory syllable were reduced to a single principal component that accounted for 85.7% of the total variation. A one-way ANOVA with the PC scores showed that spectral and temporal measurements of the introductory syllables varied significantly among leks (F_8,67_ = 12.81, *p*<0.0001; **Figure 1** **, Table S2**) and among groups of leks along the Sierra Madre Oriental (F_2,67_ = 13.32, *p*<0.0001). Regarding measurements of the shared syllable, the 13 variables were reduced to three principal components that accounted for 81% of the variation. The first PC (52.1%) was mainly explained by the peak frequency of the syllable, PC2 (18.8%) by the peak frequency of the second element, and PC3 (10%) was explained by duration, minimum frequency, and bandwidth of the syllable and their elements. A MANOVA with the PC scores also showed significant variation among leks (Wilks’ Lambda, F_8,65_ = 1.7, *P*<0.05; **Table S3**) but the variation among groups of leks was not significant (Wilks’ Lambda, F_6,122_ = 0.8, *p* = 0.06).

Results of the DFA based on the relative frequency of syllables, and the acoustic measurements of the introductory and the shared syllable showed that 100%, 70% and 82% of the individuals, respectively, were correctly classified by lek membership. **Figure 1** shows examples of the introductory and shared syllables from each lek. In the DFA with the relative frequency of syllables the first three discriminant functions recovered 74.2% of the variation (function 1: eigenvalue 360.4, 43% variance; function 2: eigenvalue 149.4, 17.7% variance; function 3: eigenvalue 116.2, 14% variance). In the DFA with acoustic measurements of the introductory syllables, the first three discriminant functions recovered 89.3% of the variation (function 1: eigenvalue 3.25, 36% variance; function 2: eigenvalue 1.18, 27% variance; function 3: eigenvalue 0.90, 17% variance). Lastly, the first two discriminant functions recovered 77.7% of the variation (function 1: eigenvalue 5.9, 67.6% variance; function 2: eigenvalue 0.88, 10.1% variance) in the DFA with acoustic measurements of the shared syllable.

### Comparison between Acoustic, Genetic and Geographic Distances

**Figure 6:**
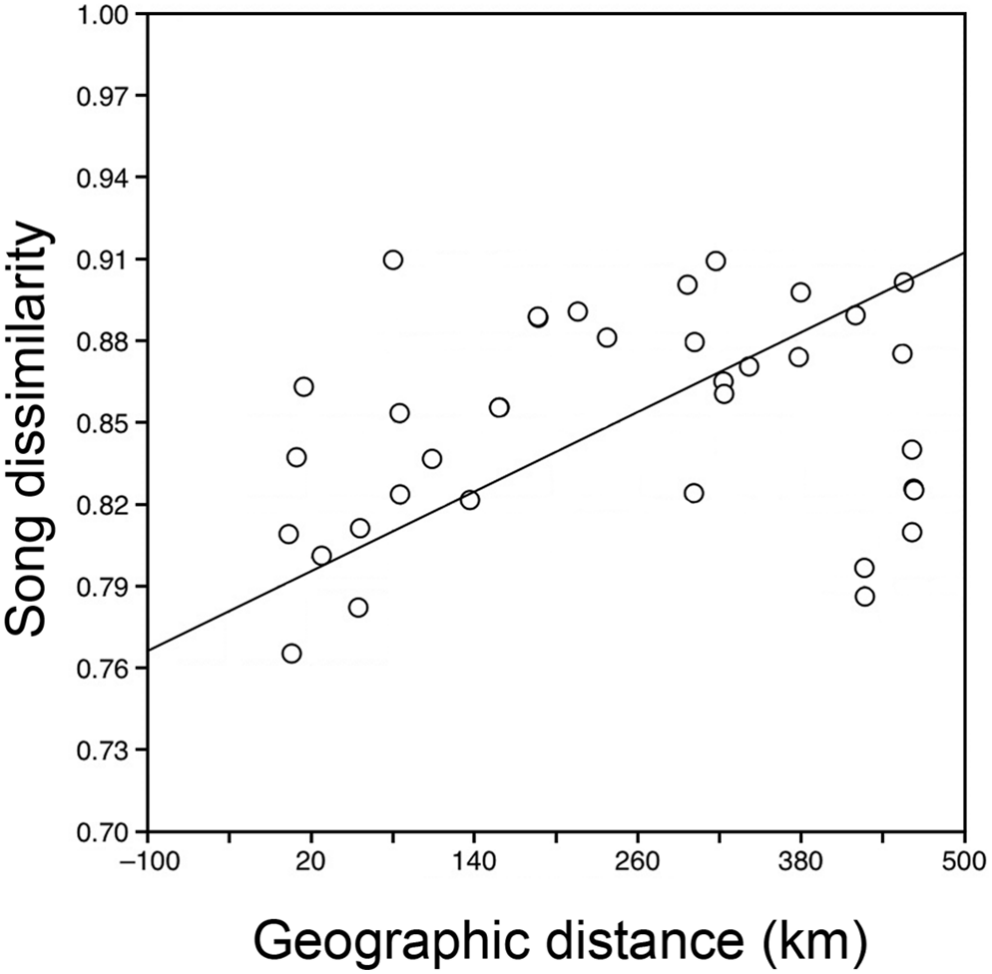
Significant correlation between acoustic and geographic distance from a Mantel test. Circles represent mean values of pairwise comparisons between Jaccard similarity coefficient between individuals among leks and geographic distance among leks. Song dissimilarity was calculated by subtracting the Jaccard similarity coefficient values from 1 to make a dissimilarity matrix; geographic distance estimated as linear distance in kilometers among leks.

We found that the correlation between R_ST_ values and Euclidean distance values of acoustic variables that differentiated *C. curvipennis* leks was not significant (Mantel test: song sharing, *r* = –0.06, *p* = 0.618; introductory syllable *r* = 0.06; *p* = 0.590; shared syllable, *r* = 0.13, *p* = 0.678). Likewise, the correlations between F_ST_ values and acoustic measures were no statistically significant (Mantel test: song sharing, *r* = –0.44, *p* = 0.992; introductory syllable *r* = –0.31; *p* = 0.917; shared syllable, *r* = 0.02, *p* = 0.570). In contrast, the correlation between acoustic and geographic distances was positive and significant when using the song sharing distance matrix (*r* = 0.24, *p* = 0.04; **Figure 6**), indicating that song sharing decreases with increased geographic distance among leks. However, the correlation was not significant when using the Euclidean distances based on measurements of the introductory syllable (*r* = 0.09, *p* = 0.835) or the shared syllable (*r* = –0.006, *p* = 0.389). Removing the effects of genetic distance did not significantly alter the positive relationship between geographic and song sharing distances (partial Mantel test, *r* = 0.24, *p* = 0.04). Finally, genetic and geographic distances were not significantly correlated (R_ST_, *r* = –0.026, *p* = 0.6; F_ST_, *r* = –0.07, *p* = 0.4).

## Discussion

### Song Divergence with Gene Flow

Our study revealed that lek members of the *curvipennis* subspecies studied here are not genetically structured along the Sierra Madre Oriental. Measures of migration rates, genetic differentiation and population structure indicated gene flow among leks and low levels of genetic structure. The relationships between acoustic and genetic distances were sharply discordant with previous results comparing acoustic and genetic data of all three wedge-tailed sabrewing subspecies. González et al. [14] showed strong positive correlations between genetic (F_ST_ and R_ST_ values) and acoustic distances (song sharing and common syllable), and the significance of these relationships was not altered when the effects of geographic distance were removed. These analyses indicate that genetically divergent subspecies shared fewer syllable types, and differed acoustically in the common syllable, independently of distance, which might be the result of long isolation after divergence in allopatry. However, the relationships between acoustic and genetic distances were not statistically significant within the *curvipennis* subspecies studied here (along the Sierra Madre Oriental). Although song traits play a potential role in increasing genetic polymorphisms and generating reproductive isolation and speciation in parapatry (or even in sympatry through sexual selection [3], [44]), our data suggest that the strong vocal divergence is not acting as a reproductive isolating barrier in the *curvipennis* subspecies; song divergence in this hummingbird species does not impede gene flow along the Sierra Madre Oriental. In contrast, both multivariate and STRUCTURE analyses of *curvipennis* male songs were congruent in detecting a strong pattern of acoustic structure with sharp boundaries between leks, where each lek had an exclusive assemblage of syllable types, and song sharing between leks was lower than within them. Also, acoustic traits of two types of comparable syllables (introductory and shared syllables) were more divergent between than within leks.

### Decoupled Patterns of Genetic and Acoustic Divergence

Both the genetic adaptation and epiphenomenon hypotheses predict that dialects do affect genetic structure (although not causally in the epiphenomenon hypothesis; [42]–[44], [46]). These hypotheses emphasize that a strong positive correlation between neutral genetic and acoustic divergence would indicate that, under certain conditions, acoustic divergence is largely the result of stochastic forces of mutation and drift [21], [60]. In contrast, the social adaptation hypothesis predicts no relationship between song dialects and the genetic structure of populations [45]. Accordingly, a comparison of genetic and acoustic divergence in the *curvipennis* subspecies yielded a non-significant correlation between genetic and acoustic distance. Studies using rapidly evolving genetic markers in a wide variety of song learners, mainly songbirds and parrots, have shown similar patterns of genetic differentiation (F_ST_), and have also failed to find genetic divergence coupled with song divergence [12], [18], [25], [61]–[64]. Therefore, our study is first to quantify the relationship between genetic and acoustic variation at the subspecies level, in taxa in which song learning has evolved independently [27]–[28]. Contrary to predictions of the long-standing hypothesis that avian dialects contribute to reproductive isolation between populations, our study adds to the growing number of studies that have incorporated rapidly evolving genetic markers such a microsatellites [12], [25], [62]–[63], [65], yet failed to find an association between acoustic and genetic variation.

The decoupled patterns between the neutral genetic and acoustic distance highlights the role that learning might play in the formation of lek-level vocal structure in wedge-tailed sabrewings. As in songbirds and parrots, song learning in hummingbirds is culturally transmitted through imitation [27], and the process of learning across generations and the creation of new songs, given the high number of syllables in the repertoire of wedge-tailed sabrewings [14], [36], may provide important sources of variation [3], [41], [67]. Theoretical work of song variation and dispersal proposed that the degree of postdispersal learning is critical in determining the degree of genetic divergence between populations with divergent songs and that song divergence could evolve at the population level under a range of conditions [67]. In addition, mathematical modeling on the maintenance of bird song dialects has shown that the combination of both low dispersal and strong assortative mating based on song promotes dialect maintenance [68]. Therefore, the extent of dispersal and the capacity of postdispersal learning are critical in determining the degree for genetic divergence between populations acoustically structured, where song divergence in predispersal learners can most likely lead to a higher genetic subdivision than song divergence in open-ended learners [61], [67]. Dispersal patterns of wedge-tailed sabrewings are not known, however, the observed geographic structure of song without genetic differentiation among lek members suggests that wedge-tailed sabrewings learn their vocalizations after dispersal, probably to facilitate territory establishment and/or the access to leks. A recent study of vocalizations of the long-billed hermit (*Phaethornis longirostris*) showed that individuals changed their song-type at leks during a four-year period, and that all song replacements occurred after a crystallized song was already produced, which indicates that song learning may well extend throughout their lifespan [31]. The sensitive phase of learning in hummingbirds is not known with certainty [31]–[32], but it might be sufficiently extended to learn their songs before or at the time of territory establishment in wedge-tailed sabrewings because we have occasionally observed, and recorded, young males lacking a territory emitting imperfect and very quiet songs at leks. Besides, song neighborhoods within leks of wedge-tailed sabrewings [37] are not static over time because new local or foreign syllables are incorporated each year, and apparently some syllables go extinct. Although nothing is known about the tempo of vocal learning in this species, our previous work indicates that males alter their singing display over time [37], suggesting that vocal learning in this species may also be open-ended, as shown for the long-billed hermit [31]. But whether wedge-tailed sabrewings learn their songs in their natal lek or not is an open question that cannot be answered with our current data.

### Founder Effects and Male-Male Competition Causing Song Divergence

Interestingly, we observed a significant correlation between song sharing and geographic distance after removing the effects of genetic distance, and the STRUCTURE analysis based on the presence/absence of syllable types data set revealed a marked pattern of song structure between leks consistent with their geographic origin (**Figure 4**), with a clear separation between leks from the northern, central, and the southern part of the distribution along the Sierra Madre Oriental. We also observed that two of the northern leks (nSMO = Ciel and GF) had a smaller number of elements and number of different elements in the introductory syllable than southern leks, but the pool of syllables (number of different syllables and number of exclusive syllables) was equally diverse in the northern and southern leks (**Table S2**), indicating that the observed geographic structure of *curvipennis* songs can not be explained by founder effects after colonization of northern areas [6], [22]–[23]. Instead, the isolation-by-distance model explains best the marked geographic pattern of song structure between leks (*K* = 3; **Figure 4**). However, as shown mathematically, the nonlinear effects of assortative mating and song learning could also give rise to the emergence of dialects when explored at a spatial scale [68]. Despite the observed geographic pattern of acoustic variation, and the vanishingly small to zero gene flow pattern in the south-to-north direction, no genetic structure was detected. Therefore, the strong differentiation of *curvipennis* songs along the Sierra Madre Oriental lend support to the idea that song sharing between lek members (or sharing songs with closest neighbor in leks with song neighborhoods [37]) confers social benefits that may increase reproductive success [25], [69] and/or reduce levels of aggression in male-male competition [66]–[67].

### Cultural Drift and Selection

Like other phenotypic traits, song evolution has been driven by a combination of selective pressures and stochastic factors [8], [70]. Song evolution could also be a consequence of genetic drift, i.e. following vicariant isolation, evolution by random changes in the mechanisms involved in vocal ontogeny and production, due to changes in the loci encoding those traits [70]. On the other hand, cultural drift refers to changes in song memes and subsequent fixation of the new variants by behavioral matching, driven by chance variation in their propagation across generations [8], [71]. Vocal learning through imitation could then generate the rapid transmission of new acoustic elements, contributing to geographic variation and dialect formation (reviewed in [41], [66], [72]). During the learning process copy imperfections can occur and, as a consequence, novel variations and the possibility of recombination and innovation of elements introduce variation in song leading to rapid signal change. Also, if males learn their songs after dispersing to the lekking sites, learning could again lead to vocal variation in the absence of genetic structure, without necessarily invoking any form of selection other than cultural selection (but see [68]). Therefore, it seems entirely possible that random cultural drift due to copy imperfections could rapidly change song traits, leading to the observed acoustic divergence in the absence of genetic divergence.

The decreased syllable sharing with increased geographic distance (isolation-by-distance) suggests that song divergence is also a result of neutral processes, where individuals within leks copy syllables randomly from each other and small differences in song accumulate along a geographical gradient due to the reduced probability of interaction among individuals from more distant leks (i.e. between geographic areas with no apparent barriers to gene flow). Then, the accumulated syllabic differences in song are probably distributed within a lek by behavioral matching between its members, which would explain why lek members share a high proportion of syllables. Regardless of the distance between leks, the song of each lek had a characteristic introductory syllable with strong differences in structure. This finding is consistent with previous observations in which members of song neighborhoods within leks shared the introductory syllable [37], a potential vocal signature related to social adaptation [73]. Besides the effects of geographic distance shaping patterns of song sharing between wedge-tailed sabrewing leks, adaptation of sound transmission by microclimate and vegetation structure can be important selection pressures of birds living in different habitats [74]–[75]. However, the studied leks were located in habitats with very similar climatic conditions and no significant relationships were found between acoustic distance measures vs. habitat-related (climate and topography) distance measures in a previous study [14]. Lastly, social selection or adaptation to social conditions (*e.g*., male-male competition), could be a selective force operating to promote convergence within wedge-tailed sabrewings leks, where song sharing among neighbors confers social benefits that could possibly increase their reproductive success [25]. Here the joint effects of social adaptation, the presence of an introductory note for signaling group membership as to decrease aggression, and female choice for novel cultural variants would generate the observed pattern of within-lek vocal variation in wedge-tailed sabrewings [36]–[37]. Under this scenario, the adaptive significance of song learning implies that it evolved to allow individuals to adapt to each other in an immediate social context (the social selection hypothesis).

In summary, our study reveals independence between acoustic and neutral genetic divergence in wedge-tailed sabrewings, contrary to predictions of neutral evolution hypotheses. Along the continuous distribution of this subspecies, with no apparent geographic barriers, genetic divergence appears to be restricted by homogenizing gene flow, and geographical structure of vocal variation corresponds with restricted south-to-north gene flow between geographic areas of the Sierra Madre Oriental. Finally, the highly structured and marked song divergence between leks suggests that the evolution of song elaboration in wedge-tailed sabrewing resulted from a combination of processes probably linked to postdispersal learning, isolation by distance, and social factors associated with male-male interactions. As these birds are seemly open-ended learners, we interpret our results as that cultural variation appears more plausible than genetic variation in modifying songs differently at different leks and that social selection is involved in inducing this divergence. With negligible geographic barriers and no behavioral checks on dispersal, little genetic differentiation has developed in wedge-tailed sabrewings along the Sierra Madre Oriental despite the apparent intense social (and sexual) selection acting on the elaborate acoustic traits (see also [65]). Although learning and cultural drift are real possibilities, there may be cultural selection reinforced by selection for imitation driving which songs can be learned, and coupled patterns of genetic and acoustic divergence could emerge when barriers become strong enough to gene flow.

## Supporting Information

Figure S1 Measurements taken from the (A) introductory, and (B) shared syllable emitted by every recorded individual. Numbers in (B) refer to three elements where the same measures as the complete syllable were taken. A fragment of a song bout is shown in (C) indicating the introductory syllable with an asterisk and the shared syllable with arrows.
(TIF)

Table S1 Localities, geographic location and altitude of wedge-tailed sabrewing sampled leks. Regions correspond to north, central and south of the Sierra Madre Oriental (nSMO, cSMO and sSMO).
(DOC)

Table S2 Mean ± SD of spectral and temporal measurements of introductory syllables across leks of wedge-tailed sabrewings. N = number of individuals, n = number of songs.
(DOC)

Table S3 Mean ± SD of spectral and temporal measurements of the shared syllable and three of its elements, across leks of wedge-tailed sabrewings (see Figure S1). N = number of individuals, n = number of songs.
(DOC)

Audio S1 Song of El Cielo lek.
(WAV)

Audio S2 Song of Gómez Farías lek.
(WAV)

Audio S3 Song of El Naranjo lek.
(WAV)

Audio S4 Song of Aquismón lek.
(WAV)

Audio S5 Song of Xilitla lek.
(WAV)

Audio S6 Song of Cuetzalan lek.
(WAV)

Audio S7 Song of Macuiltépetl lek.
(WAV)

Audio S8 Song of La Orduña lek.
(WAV)

Audio S9 Song of Ursulo Galván lek.
(WAV)
