# Supplementary figures and images for: Acoustic Divergence with Gene Flow in a Lekking Hummingbird with Complex Songs
Source: PLoS One. 2014 Oct 1;9(10):e109241. doi: 10.1371/journal.pone.0109241 (PMC4182805; doi:10.1371/journal.pone.0109241)

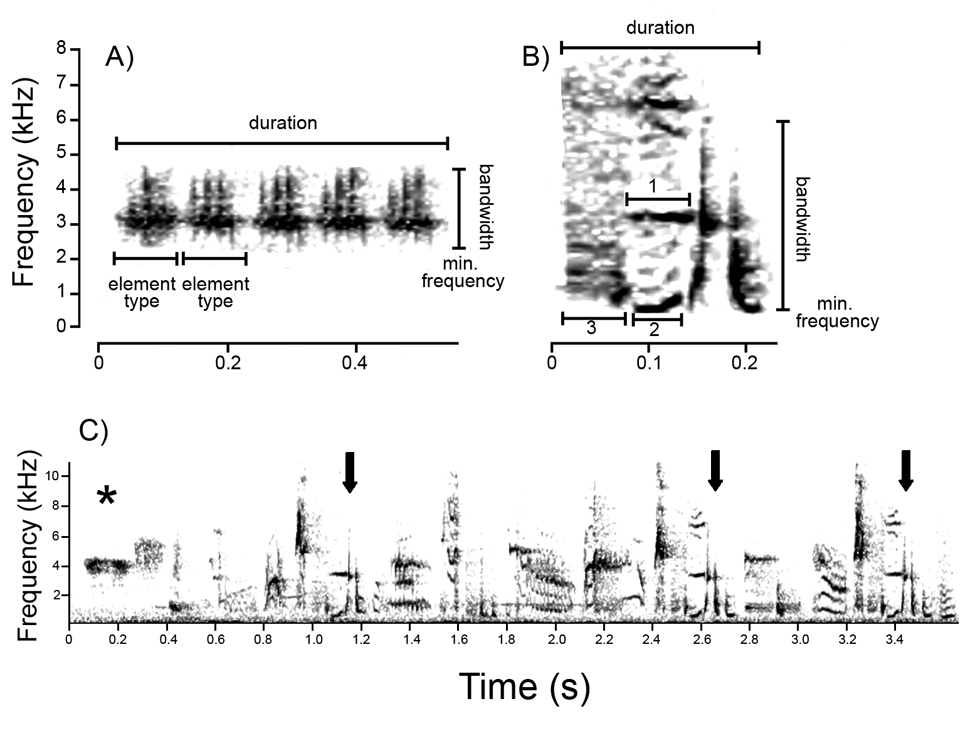

Supplement: Figure S1 — Measurements taken from the (A) introductory, and (B) shared syllable emitted by every recorded individual. Numbers in (B) refer to three elements where the same measures as the complete syllable were taken. A fragment of a song bout is shown in (C) indicating the introductory syllable with an asterisk and the shared syllable with arrows. (TIF) [file pone.0109241.s001.tif]
