# Supplementary material for: Acoustic Divergence with Gene Flow in a Lekking Hummingbird with Complex Songs
Source: PLoS One. 2014 Oct 1;9(10):e109241. doi: 10.1371/journal.pone.0109241 (PMC4182805; doi:10.1371/journal.pone.0109241)
Supplement: Table S1 — Localities, geographic location and altitude of wedge-tailed sabrewing sampled leks. Regions correspond to north, central and south of the Sierra Madre Oriental (nSMO, cSMO and sSMO). (DOC) [file pone.0109241.s002.doc]

**Table S1. Localities, geographic location and altitude of wedge-tailed sabrewing sampled leks.** Regions correspond to north, central and south of the Sierra Madre Oriental (nSMO, cSMO and sSMO).

|  |  |  |  |  |
| --- | --- | --- | --- | --- |
| **Location** | **Region** | **Latitude**  **(N)** | **Longitude**  **(W)** | **Altitude**  **(m asl)** |
| 1. El Cielo, Tamaulipas (Ciel) | nSMO | 25º 30´ 33.66´´ | 99º 12´ 21.40´´ | 943 |
| 2. Gomez Farías, Tamaulipas (GF) | nSMO | 23º 30´ 58.26´´ | 99º 10´ 06.52´´ | 564 |
| 3. El Naranjo, San Luis Potosí (Nar) | nSMO | 22º 34´ 33.33´´ | 99º 21´ 11.80´´ | 270 |
| 4. Aquismón, San Luis Potosí (Aqm) | cSMO | 21º 37´ 30.87´´ | 99º 10´ 12.52´´ | 378 |
| 5. Xilitla, San Luis Potosí (Xil) | cSMO | 21º 22´ 39.50´´ | 98º 59´ 35.77´´ | 637 |
| 6. Cuetzalan, Puebla (Cuet) | sSMO | 20º 00´ 49.14´´ | 97º 30´ 21.27´´ | 906 |
| 7. Macuiltépetl, Veracruz (Mac) | sSMO | 19º 32´ 50.51´´ | 96º 55´ 12.45´´ | 1500 |
| 8. La Orduña, Veracruz (Ord) | sSMO | 19º 27´ 50.94´´ | 96º 56´ 13.05´´ | 1190 |
| 9. Ursulo Galván, Veracruz (UG) | sSMO | 19º 25´ 31.48´´ | 96º 58´ 35.20´´ | 1200 |
