# Supplementary material for: Acoustic Divergence with Gene Flow in a Lekking Hummingbird with Complex Songs
Source: PLoS One. 2014 Oct 1;9(10):e109241. doi: 10.1371/journal.pone.0109241 (PMC4182805; doi:10.1371/journal.pone.0109241)
Supplement: Table S2 — Mean ± SD of spectral and temporal measurements of introductory syllables across leks of wedge-tailed sabrewings. N = number of individuals, n = number of songs. (DOC) [file pone.0109241.s003.doc]

**Table S2. Syllables and mean ± SD of spectral and temporal measurements of introductory syllables across leks of wedge-tailed sabrewings.**

|  |  |  |  |  |  |  |  |  |  |  |
| --- | --- | --- | --- | --- | --- | --- | --- | --- | --- | --- |
| **Lek** | **N** | **n** | **No. different syllables** | **No. exclusive syllables** | **Duration (s)** | **Min. frequency (kHz)** | **Bandwidth (kHz)** | **Peak Frequency (kHz)** | **No. elements** | **No. different elements** |
| 1. Ciel | 13 | 143 | 89 | 42 | 0.4 ± 0.12 | 2.97 ± 0.5 | 2.19 ± 0.87 | 4.09 ± 0.35 | 3.85 ± 1.57 | 1.61 ± 1.04 |
| 2. GF | 5 | 25 | 41 | 17 | 0.305 ± 0.04 | 4.43 ± 0.19 | 2.12 ± 0.22 | 5.51 ± 1.12 | 1 ± 0 | 1 ± 0 |
| 3. Nar | 4 | 17 | 51 | 18 | 0.55 ± 0.07 | 3.15 ± 0.34 | 3.2 ± 0.5 | 4.31 ± 0.09 | 7 ± 1.09 | 2 ± 0 |
| 4. Aqm | 5 | 21 | 58 | 23 | 0.74 ± 0.23 | 2.66 ± 0.53 | 3.1 ± 0.54 | 3.77 ± 0.5 | 6.6 ± 1.35 | 2 ± 0 |
| 5. Xil | 7 | 33 | 50 | 21 | 0.73 ± 0.15 | 2.81 ± 0.49 | 3.25 ± 0.69 | 4.12 ± 0.32 | 9.28 ± 1.98 | 2.86 ± 0.38 |
| 6. Cuet | 3 | 27 | 33 | 12 | 0.76 ± 0.3 | 3.28 ± 0.46 | 2.51 ± 0.62 | 3.96 ± 0 | 5.67 ± 0.58 | 2.67 ± 0.58 |
| 7. Mac | 3 | 43 | 100 | 54 | 0.86 ± 0.61 | 2.47 ± 1.22 | 2.81 ± 0.92 | 4.43 ± 0.78 | 11.5 ± 7.23 | 1.75 ± 0.5 |
| 8. Ord | 10 | 149 | 30 | 14 | 0.54 ± 0.19 | 2.73 ± 0.6 | 2.78 ± 1.1 | 4.24 ± 0.21 | 5.75 ± 2.41 | 2.12 ± 0.34 |
| 9. UG | 6 | 42 | 41 | 10 | 0.55 ± 0.09 | 3.23 ± 0.48 | 2.1 ± 0.58 | 4.11 ± 0.46 | 4.6 ± 0.84 | 2.2 ± 0.42 |

N = number of individuals, n = number of songs.
