# Supplementary material for: Acoustic Divergence with Gene Flow in a Lekking Hummingbird with Complex Songs
Source: PLoS One. 2014 Oct 1;9(10):e109241. doi: 10.1371/journal.pone.0109241 (PMC4182805; doi:10.1371/journal.pone.0109241)
Supplement: Table S3 — Mean ± SD of spectral and temporal measurements of the shared syllable and three of its elements, across leks of wedge-tailed sabrewings (see Figure S1). N = number of individuals, n = number of songs. (DOC) [file pone.0109241.s004.doc]

**Table S3. Mean ± SD of spectral and temporal measurements of the shared syllable and three of its elements, across leks of wedge-tailed sabrewings (see Figure S1).**

|  |  |  |  | |  | |  | |  | |  | | |  |  |  | |  |  | |  |  | |  | |
| --- | --- | --- | --- | --- | --- | --- | --- | --- | --- | --- | --- | --- | --- | --- | --- | --- | --- | --- | --- | --- | --- | --- | --- | --- | --- |
| **Lek** | **N** | **n** | **Duration (s)** | | **Minimum frequency (kHz)** | | **Bandwidth (kHz)** | | **Peak frequency (kHz)** | | **Duration (s)** | | | **Minimum frequency (kHz)** | **Bandwidth (kHz)** | **Peak frequency (kHz)** | | **Duration (s)** | **Duration (s)** | | **Minimum frequency (kHz)** | **Bandwidth (kHz)** | | **Peak frequency (kHz)** | |
|  |  |  | |  | | **element 1** | |  |  |  | |  | **element 2** | | | |  | **element 3** |  | **complete syllable** | | |  | |  |
| 1. Ciel | 13 | 143 | 0.07 ± 0.00 | | 3.17 ± 0.05 | | 0.37 ± 0.05 | | 3.30 ± 0.11 | | 0.05 ± 0.00 | | | 0.40 ± 0.03 | 0.56 ± 0.06 | 0.78 ± 0.13 | | 0.07 ± 0.00 | 0.20 ± 0.00 | | 0.39 ± 0.04 | 6.10 ± 0.10 | | 4.56 ± 0.77 | |
| 2. GF | 5 | 25 | 0.07 ± 0.01 | | 3.36 ± 0.05 | | 0.40 ± 0.05 | | 3.56 ± 0.10 | | 0.05 ± 0.01 | | | 0.39 ± 0.05 | 0.64 ± 0.06 | 0.77 ± 0.12 | | 0.06 ± 0.00 | 0.20 ± 0.01 | | 0.37 ± 0.05 | 6.52 ± 0.08 | | 4.75 ± 1.25 | |
| 3. Nar | 4 | 17 | 0.07 ± 0.00 | | 2.89 ± 0.05 | | 0.43 ± 0.07 | | 3.16 ± 0.16 | | 0.05 ± 0.01 | | | 0.37 ± 0.01 | 0.57 ± 0.04 | 0.73 ± 0.15 | | 0.07 ± 0.00 | 0.21 ± 0.00 | | 0.37 ± 0.02 | 5.65 ± 0.15 | | 3.06 ± 1.24 | |
| 4. Aqm | 5 | 21 | 0.07 ± 0.00 | | 2.89 ± 0.06 | | 0.31 ± 0.06 | | 3.13 ± 0.11 | | 0.05 ± 0.00 | | | 0.43 ± 0.02 | 0.57 ± 0.07 | 0.72 ± 0.22 | | 0.08 ± 0.00 | 0.21 ± 0.01 | | 0.41 ± 0.03 | 5.42 ± 0.29 | | 4.55 ± 0.99 | |
| 5. Xil | 7 | 33 | 0.07 ± 0.00 | | 2.97 ± 0.07 | | 0.43 ± 0.09 | | 3.19 ± 0.17 | | 0.05 ± 0.00 | | | 0.43 ± 0.06 | 0.59 ± 0.07 | 0.69 ± 0.26 | | 0.07 ± 0.00 | 0.20 ± 0.00 | | 0.42 ± 0.05 | 5.67 ± 0.11 | | 3.87 ± 1.45 | |
| 6. Cuet | 3 | 27 | 0.07 ± 0.00 | | 3.01 ± 0.18 | | 0.32 ±0.06 | | 3.13 ± 0.22 | | 0.05 ± 0.00 | | | 0.47 ± 0.04 | 0.57 ± 0.00 | 0.95 ± 0.09 | | 0.07 ± 0.00 | 0.21 ± 0.00 | | 0.46 ± 0.02 | 5.72 ± 0.20 | | 3.87 ± 2.03 | |
| 7. Mac | 3 | 43 | 0.07 ± 0.00 | | 3.26 ± 0.11 | | 0.41 ± 0.05 | | 3.47 ± 0.08 | | 0.05 ± 0.00 | | | 0.39 ± 0.06 | 0.63 ± 0.11 | 0.80 ± 0.11 | | 0.07 ± 0.01 | 0.20 ± 0.01 | | 0.39 ± 0.07 | 6.32 ± 0.19 | | 4.28 ± 1.80 | |
| 8. Ord | 10 | 149 | 0.07 ± 0.00 | | 3.14 ± 0.10 | | 0.38 ± 0.05 | | 3.34 ± 0.13 | | 0.05 ± 0.00 | | | 0.41 ± 0.03 | 0.63 ± 0.05 | 0.85 ± 0.19 | | 0.07 ± 0.00 | 0.20 ± 0.00 | | 0.40 ± 0.04 | 6.09 ± 0.17 | | 4.29 ± 1.36 | |
| 9. UG | 6 | 42 | 0.07 ± 0.01 | | 3.09 ± 0.11 | | 0.36 ± 0.06 | | 3.24 ± 0.13 | | 0.05 ± 0.00 | | | 0.41 ± 0.04 | 0.65 ± 0.07 | 0.75 ± 0.19 | | 0.07 ± 0.00 | 0.20 ± 0.00 | | 0.40 ± 0.03 | 5.89 ± 0.19 | | 3.79 ± 1.79 | |

N = number of individuals, n = number of songs.
